# Fascinations of Science

**Published:** 1875-09

**Authors:** 


Fascinations of Science.—Mark Twain, in the *Atlantic Monthly* for August, says: The Mississippi river, between Cairo and New Orleans, was twelve hundred and fifteen miles long one hundred and seventy-six years ago. It was eleven hundred and eighty after the cut-off of 1722. It was one thousand and forty after the American Bend cut-off (some sixteen or seventeen years ago). It has lost sixty-seven miles since. Consequently, it is only nine hundred and seventy-three miles at present. Now, if I wanted to be one of those ponderous scientific people, and "let on" to prove what had occurred in the remotest past by what had occurred in a given time in the recent past, or what will occur in the far future by what has occurred in late years, what an opportunity is here! Geology never had such a chance, nor such exact *data* to argue from! Nor "development of species," either! Glacial epochs are great things, but they are vague—vague. Please observe: In the space of one hundred and seventy-six years the Lower Mississippi has shortened itself two hundred and forty-two miles. That is an average of a trifle over one mile and a third per year. Therefore, any calm person, who is not blind or idiotic, can see that in the Old Oolitic Silurian Period, just a million years ago next November, the Lower Mississippi river was upward of one million three hundred thousand miles long, and stuck out over the Gulf of Mexico like a fishing-rod. And by the same token any person can see that seven hundred and forty-two years from now the Lower Mississippi will be only a mile and three-quarters long, and Cairo and New Orleans will have joined their streets together, and be plodding comfortably along under a single mayor and a mutual board of aldermen. There is something fascinating about science. One gets such wholesale returns of conjecture out of such a trifling investment of fact.—*Phar. Gaz.*

Dr. Fleetwood Churchill, of Dublin, the well known obstetrician, has resigned practice, and will reside for the future in North of Ireland. He was President of Col. Physicians in 1857–68, and has presented about one thousand volumes, consisting mostly of works on obstetric medicine and the diseases of children, to the library of that institution. The college have arranged to have a life-size portrait of Dr. Churchill, painted in oil by Mr. Jones, R. H. A., placed in the Examination Hall, and an illuminated address will shortly be presented to him by the Fellows of the college, assuring him of the respect and esteem in which he is regarded by each member of that body.—*Lancet.*

